# Neutrophils and Macrophages as Targets for Development of Nanotherapeutics in Inflammatory Diseases

**DOI:** 10.3390/pharmaceutics12121222

**Published:** 2020-12-17

**Authors:** Yujie Su, Jin Gao, Puneet Kaur, Zhenjia Wang

**Affiliations:** 1 Department of Pharmaceutical Sciences, College of Pharmacy and Pharmaceutical Sciences, Washington State University, Spokane, WA 99202, USA; yujie.su@wsu.edu (Y.S.); jin.gao3@wsu.edu (J.G.); 2 College of Pharmacy and Pharmaceutical Sciences, Washington State University, Spokane, WA 99202, USA; p.kaur@wsu.edu

**Keywords:** nanomedicine, neutrophils, macrophages, cell targeting, interactions of nanoparticles and cells, inflammatory diseases

## Abstract

Neutrophils and macrophages are major components of innate systems, playing central roles in inflammation responses to infections and tissue injury. If they are out of control, inflammation responses can cause the pathogenesis of a wide range of diseases, such as inflammatory disorders and autoimmune diseases. Precisely regulating the functions of neutrophils and macrophages in vivo is a potential strategy to develop immunotherapies to treat inflammatory diseases. Advances in nanotechnology have enabled us to design nanoparticles capable of targeting neutrophils or macrophages in vivo. This review discusses the current status of how nanoparticles specifically target neutrophils or macrophages and how they manipulate leukocyte functions to inhibit their activation for inflammation resolution or to restore their defense ability for pathogen clearance. Finally, we present a novel concept of hijacking leukocytes to deliver nanotherapeutics across the blood vessel barrier. This review highlights the challenges and opportunities in developing nanotherapeutics to target leukocytes for improved treatment of inflammatory diseases.

## 1. Introduction

Inflammation is a host protection response to infections and tissue injury [1]. This process includes recognition, removal of harmful foreign objects and repair of damaged tissues, and it is a tightly regulated program which is essential for maintaining homeostasis. However, if this process is out of control, inflammation can cause tissue injury leading to various diseases. As an example, nonresolving inflammation results in tissue damage and metabolic disorders, thus, impairing organ functions. Sometimes, inflammation initiates tissue repair via the formation of fibrosis to maintain structural integrity, but this further aggravates tissue dysfunction [2]. Studies have revealed that inflammation is involved with the pathogenesis of diverse diseases, including infectious diseases [3], autoimmune disorders, atherosclerosis, neurodegeneration [4], metabolic syndromes, cancer and other chronic diseases [5].

Inflammation is involved with the activation and movement of neutrophils and macrophages. Neutrophils are also called polymorphonuclear cells and are the most abundant circulating white blood cells in humans with 50–70% of leukocytes being neutrophils [6]. Neutrophils have a short lifetime and are replenished from the bone marrow [7]. Neutrophils play a central role in the acute inflammation response. In case of tissue injury or pathogen invasion, neutrophils are rapidly activated in the bloodstream and are the first leukocytes to arrive at injured lesions. Infiltrated neutrophils eliminate pathogens and also initiate inflammatory cascades [8].

Macrophages are tissue resident immune cells and can be generated from monocytes in the blood after their activation and tissue infiltration. Macrophages also play a major role in regulating inflammation responses via the transition of their phenotypes. There are two phenotypes of macrophages. Typically, macrophages polarize to become a classically activated (M1) phenotype which promotes inflammation, but an activated phenotype (M2) terminates inflammation for tissue repair [9]. It has been shown that neutrophils and macrophages orchestrate with adaptive immune cells to regulate immune systems achieving a balance of inflammation and its resolution. Once danger molecules have been cleared, dead neutrophils can be eliminated by macrophages to return to homeostasis [10]. If this process fails, pathological conditions occur, resulting in uncontrolled cytokines release, tissue damage and immune disorders. This intricate role of neutrophils and macrophages in inflammation responses may be a novel target for developing therapeutic modalities to treat a wide range of inflammatory diseases.

Although neutrophils [11,12] and macrophages [13] are considered as therapeutic targets, specifically targeting them is still challenging because most drugs do not possess targeting properties. In addition, neutrophils and macrophages are involved in complex immune systems, thus, the non-selective actions of these innate immune cells may cause the systemic toxicity [14,15]. Nanomedicine for targeted drug delivery has received significant attention in recent decades because advances in nanotechnology enable us to rationally engineer nanomaterials. Nanoparticles have large surface areas and can be bioengineered for drug targeting and controlled drug release at the correct tissues in contrast to free drugs. Targeting neutrophils or macrophages using nanoparticles seems to have inherent advantages based on their host front-line defense and preference to interact with nanoparticles [16,17]. Nanoparticles can easily bind to or be taken up by immune cells via phagocytosis. Therefore, nanotherapeutics may precisely regulate the functions of neutrophils and macrophages.

In this review, we first provide a general overview of neutrophil and macrophage biology and their roles as potential therapeutic targets in inflammatory diseases. We then summarize and discuss immuno-targeting to neutrophils and macrophages by nanoparticles; these interactions are crucial for developments of nanoparticle-based immunotherapies. We illustrate an emerging nanomedicine that specifically targets neutrophils or macrophages for treating inflammation-associated diseases and regulating immune responses. Finally, we highlight some strategies for hijacking neutrophils and macrophages by nanoparticles to overcome biological barriers in treating inflammatory diseases and cancer.

## 2. Inflammation Responses

Neutrophils and macrophages originate from hematopoietic stem cells in the bone marrow. The differentiation and maturation of neutrophils and macrophages are complex processes, and they are both major contributors in inflammatory responses. When the host senses danger signals, endothelium and tissue resident sentinel cells (macrophages) regulate the production of neutrophils and monocytes via cytokines, such as granulocyte macrophage-colony stimulating factor (GM-CSF). Neutrophils are then released from the bone marrow into the circulation to chase danger signals. Later, monocytes infiltrate into tissues and subsequently differentiate to macrophages in order to phagocytose dead neutrophils and other dead cells for tissue repair. These movements of neutrophils and monocytes are strongly regulated through adhesion molecules (selectins, cadherins, and integrins) between leukocytes and the inflamed endothelium, and are followed by extravasation into local tissues via chemotaxis [18].

Once on site, neutrophils promptly perform their duty to kill pathogens via phagocytosis and granule secretion. The processes include the release of reactive oxygen species (ROS), hydrolytic enzymes and other antimicrobial proteins/peptides, chemokines, cytokines and lipid mediators [19]. Another feature of neutrophils is neutrophil extracellular traps (NETs) which are comprised of a complex of chromatin filaments (DNA) and neutrophil-derived antimicrobial molecules (histones and proteases) [20]. The purpose of NETs is to kill pathogens (such as bacteria), but studies have shown that they cause proinflammatory responses, including overwhelming activation of neutrophils and monocytes. Persistent and intensive inflammatory responses may lead to the long-term production of proteolytic enzymes that results in tissue damage.

By contrast, macrophages are distributed in most tissues [21,22]. Macrophages are immune sentinel cells that are capable of responding to tissue injury and pathogen invasion [23]. In addition, extravasated monocytes are a vital source of macrophages for homeostasis and inflammation responses. Macrophages are highly plastic cells and differentiate with different functions in response to the changes in the environment. During inflammation, macrophages polarize to become an M1 phenotype that produces pro-inflammatory cytokines and chemokines (TNF-α, IL1-β, IL-12, etc.) and promote adaptive immunity [24]. Conversely, an M2 phenotype is involved in the resolution of inflammation for tissue repair and remodeling by anti-inflammation cytokines, chemokines and growth factors (IL-10, TGF-β, CCL18 etc.) [25]. Such phenotypic shift is determined by an M1/M2 ratio which is temporal and spatial, therefore, this ratio is a measurable factor to evaluate inflammation resolution.

**Figure 1:**
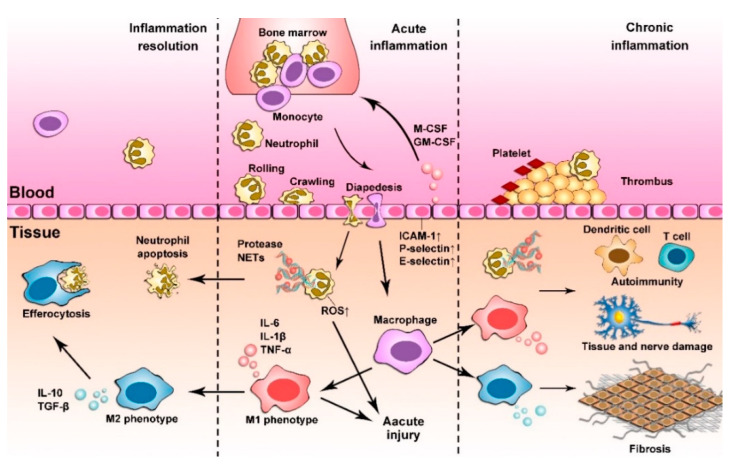
Neutrophils and macrophages regulate inflammatory responses. In acute inflammation, neutrophils and monocytes are quickly released from the bone marrow and transmigrate into infected or injured tissues. These processes are regulated by macrophage colony-stimulating factor (M-CSF) and granulocyte macrophage-colony stimulating factor (GM-CSF). Monocytes differentiate in the tissues to become macrophages and may polarize to an M1 phenotype and release pro-inflammatory factors. Activated neutrophils secrete neutrophil extracellular traps (NETs) and proteases that are also involved in the functions of M1 macrophages during acute inflammation. In the phase of inflammation resolution, NETs and proteases are degraded and neutrophils become apoptotic, while macrophages efferocytose neutrophils and dead tissues. In addition, there is a macrophage transition from M1 to M2 during resolution of inflammation. If resolution of acute inflammation fails, persistent activation of neutrophils and macrophages mediates the pathogenesis of a wide range of vascular diseases. In a chronic inflammation phase, neutrophils and macrophages activate and regulate platelets and adaptive immunity to form a new homeostasis phase that leads to inevitable tissue damage and remodeling.

Neutrophils and macrophages are highly heterogeneous. Their phenotypes are strongly associated with specific diseases and ageing. Recent studies have demonstrated that ageing is the cause of neutrophil heterogeneity and dysfunction [26] and this is also the case for macrophages [27]. Advancements in understanding the complex networks of neutrophils and macrophages have shown that neutrophils [28] and macrophages [29] are key regulators in metabolic diseases, autoimmune diseases and inflammatory disorder diseases. The functions of neutrophils and macrophages in the inflammatory response are summarized in Figure 1.

### 2.1. Acute Inflammation

Acute inflammation is characterized by the fast recruitment of leukocytes and production of inflammatory mediators after infection or injury. This acute phase is short-lived (days to weeks) and it is sometimes self-limited to allow immune cells to eliminate hazards whilst avoiding excessive tissue damage. Dysfunction of neutrophils and macrophages may cause acute inflammatory diseases, such as sepsis [30] and stroke [4].

Sepsis is a life-threatening systemic organ dysfunction caused by a dysregulated host response to infection. Studies have shown that paralysis of neutrophils is a key cause of sepsis. Neutrophils are inefficient at clearing pathogens [31] or accumulation in infected tissues [32]. Even worse, they continuously release proteolytic enzymes that induce systemic tissue damage and multiorgan failure [33]. Malfunctions of neutrophils cause the loss of neutrophils as “fighters” to attack pathogens in sepsis. Macrophages have a different response in sepsis. Macrophages differentiate to the M2 phenotype and develop the lipopolysaccharide (LPS) resistance [34], thus suppressing adaptive immune responses.

Ischemic stroke is a severe acute brain disease. Ischemia is an event in which blood vessels are occluded in the brain, leading to the sudden disruption of blood circulation. Reperfusion is a clinical tool to stimulate blood flow back to the brain, but this process causes brain damage. Ischemia/reperfusion (I/R) injury in the brain is strongly associated with acute inflammation. In ischemia, neutrophils are quickly recruited to ischemic sites [35], and platelets interact with activated neutrophils to increase vessel occlusion [36]. Reperfusion causes an increase in oxygen in the tissue which generates more ROS, leading to tissue damage [37]. Depletion of neutrophils has been shown to provide benefits in cases of ischemic stroke, suggesting that neutrophils play a central role in ischemic stroke [36]. Macrophages also contribute to ischemic stroke. The resident macrophages in the brain (termed microglial cells) are activated within minutes of ischemia onset and produce a plethora of proinflammatory mediators, including IL-1β and TNF-α, which exacerbate tissue damage [38,39]. However, peripheral monocytes/macrophages contribute to the later stages of ischemic stroke. They infiltrate into the lesion site peaking at 3 to 7 days. These infiltrated macrophages demonstrate both harmful roles and beneficial roles in ischemic stroke depending upon the timing of post-stroke and the polarization states of macrophages [40].

### 2.2. Chronic Inflammation

Inflammation could become chronic when it persists for a long time (months or years) and the host enters a new self-sustaining stage. Chronic inflammation is an unresolving inflammation response caused by persistent inflammatory stimuli. The host reaches a new homeostasis level, but it completely fails in resolution of inflammation. Chronic inflammation underlies many diseases, and studies have shown that dysfunction of neutrophils [11] and macrophages [41] play a major role in contributing to chronic inflammation.

Low-grade inflammation is identified as a pathogenesis in insulin-resistance obesity and type 2 diabetes [42]. Swollen and massive adiposes in obesity represent the onset of stress signaling pathways and increased death of adipocytes; their death offers a sustainable inflammatory environment. It has been found that macrophage markers are significantly increased in the adipose tissues of obese animals compared to those in lean animals [43]. Infiltrated macrophages are activated, and the majority are of the M1 phenotype, releasing pro-inflammatory cytokines. This transition causes insulin resistance. Neutrophils are also recruited to adipose tissues in obesity [44]. It is interesting that neutrophils release elastases that contribute to the transition of M1 macrophages. In addition, increased oxidative stress by neutrophils causes insulin signaling attenuation [45].

Neutrophils and macrophages are also involved in chronic pulmonary diseases. The M2 phenotype of macrophages produces TGF-β/IL-10 that remodels lung tissues, leading to lung fibrosis [46,47]. Neutrophils and M1 macrophages are also accomplices that instigate inflammation and tissue injury. They secrete pro-inflammatory mediators (such as TNF-α, IL-1β and NO), which increase DNA damage and mucus secretion to accelerate airway remodeling [48,49]. Recent studies have shown that the polarization of macrophages is strongly associated with the severity of chronic obstruction pulmonary disease (COPD). The percentages of M1 and M2 are significantly increased in COPD patients compared to healthy people [50]. This study indicates that M1 and M2 may be biomarkers for diagnosing COPD.

An autoimmune disease is a condition in which the host immune system mistakenly attacks itself. For example, neutrophil derived NETs contain plenty of DNA and proteins in the bloodstream, and they can be considered to be autoantigens that activate dendritic cells [51]. Inhibition of signaling pathways of NETs has been shown to elicit a reduction in lupus severity in mouse models [52], indicating that NETs are a pathogenesis of autoimmune diseases. Furthermore, macrophages transform to the M1 phenotype, promoting the antigen presentation to assist T cells which underlies autoimmune disorders.

It has been shown that ageing is strongly linked to chronic inflammation, named “inflammageing”. This inflammageing is involved with the senescence of neutrophils [53,54] and macrophages [55]. Neurodegenerative diseases are a representative example in which age-associated aggregated proteins in neurons induce the inflammation response. Microglia (macrophages in the brain) in Alzheimer’s disease (AD) patients secrete more pro-inflammatory cytokines [56] and have defects in phagocytosis of β-amyloid (Aβ) [57]. Neutrophils are found to accumulate in the brain and release NETs around Aβ sites, leading to negative outcomes [58].

Identification of neutrophils and macrophages in different phenotypes and functions linked to inflammation-associated diseases provides potential targets for pharmacological interventions. However, current technologies lack the specificity and targeting features to precisely manipulate neutrophils or macrophages in situ. The reasons for this are that neutrophils and macrophages have similar surface markers, and it is challenging to find a time window to target them without affecting the systemic immune functions of the host. Development of new and novel technologies and approaches are needed.

## 3. Immuno-Targeting by Nanoparticles

Nanotechnology has changed biomedical research and medicine because drug delivery systems based on nanotechnology have the potential to transform traditional medicine into precision medicine and nanomedicine [59,60,61,62,63]. While nanoparticle-based therapeutics have been studied in cancer for improved tumor targeting, the interactions between nanoparticles and immune systems are infantile and interesting areas because blood immune cells represent the first line of contact for intravenously administered nanoparticles. Understanding the interactions between nanoparticles and immune cells is important to develop immunotherapies and anti-inflammation therapies using nanotechnology.

### 3.1. Nanoparticle Passive Targeting Immune Cells

Neutrophils account for 50–70% of circulating leukocytes in humans [6]. Neutrophils are involved with the phagocytosis of pathogens and exogenous particles [64]. Nanoparticles are inevitably tagged by plasma proteins and complements when they circulate in the blood [65,66]. These features may allow neutrophils to easily and frequently come into contact with administrated nanoparticles.

Macrophages are the main components in various tissues and organs [67] and they are also present close to blood vessels [68]. Perivascular macrophages exist in both healthy [69] and diseased [70] tissues. The locations of macrophages control nanoparticle permeation to tissues, even though the nanoparticles have crossed a blood vessel layer. Such tissue structures have been observed in the brain [71], pancreas [72], kidney [73] and testis [74]. A recent study has shown that even in tumor tissues 70.4% of tumor blood vessels contained perivascular tumor-associated macrophages (TAMs) [75]. When nanoparticles were delivered to tumor tissues, nanoparticle uptake by TAMs was seven to 38 times higher than that by tumor cells, regardless of whether nanoparticles had active targeting ligands or not.

In fact, physicochemical properties of nanoparticles affect their uptake by immune cells to a large extent. Nanotechnology enables us to design nanoparticles with different sizes, charges, shapes, and surface chemistry. Understanding how these parameters affect the interactions of nanoparticles with neutrophils or macrophages is critical for developments in nanoparticle-based immunotherapy and optimization of nanoparticle cell targeting.

Studies of size-dependent nanoparticle uptake revealed that neutrophils more efficiently internalized large nanoparticles than small ones in the range of 20 nm to 200 nm [76]. This is an interesting observation that is different from the design of smaller nanoparticles for improved tumor therapies based on enhanced permeability and the retention effect present in tumor microenvironments [77,78].

The nanoparticle’s shape is another factor that influences nanoparticle phagocytosis. A recent study showed that neutrophils preferentially phagocytosed elongated particles compared to macrophages [79]. This selectivity of nanoparticle uptake was related to the higher motility and lower energy barrier of actin transformation in neutrophils when they engulfed elongated particles. Such preference of nanoparticle uptake may offer a method to selectively target neutrophils in vivo.

The surface chemistry of nanoparticles is also important in regulating neutrophil nanoparticle uptake. Bisso et al. [76] found that human serum reduced neutrophil uptake of poly(styrene) nanoparticles and liposomes, but increased uptake of poly(lactic-co-glycolic acid) (PLGA). However, the molecular mechanism responsible is not clear. In addition, PEGylation is used to increase nanoparticle circulation time, but a recent study showed that applying a PEG coating to nanoparticles enhanced neutrophil phagocytosis when nanoparticles were in serum. This observation may be linked to particular human complement proteins [80].

Similar to neutrophils, macrophages prefer internalization of large nanoparticles compared to small nanoparticles [81]. The shape of nanoparticles can also influence their uptake by macrophages. This shape dependence may be associated with actin transformation on the cell membrane [82] and the hydrodynamics of nanoparticles [83]. Size- and shape-dependent nanoparticle uptake has been heavily studied [82,84], but the molecular mechanism still remains controversial and further elucidation is needed.

The surface charge and coating of nanoparticles determine their hydrophobicity and steric effect. These features may dramatically affect nanoparticle uptake because protein adsorption to nanoparticles governs the trafficking of nanoparticles in vivo [85,86]. For example, highly charged nanoparticles are more easily taken up by macrophages compared to neutral nanoparticles [81,87]. This is because there are strong electrostatic interactions between nanoparticles with cell membranes or plasma proteins after nanoparticles are intravenously administered. The complement proteins adsorbed to the nanoparticle surface may amplify the phagocytosis of macrophages.

Elasticity is also a key parameter known to modulate macrophage phagocytosis. Roberto et al. [88] chose the elasticity of nanoparticles from 100 kPa (soft) to 10 MPa (rigid), revealing that soft nanoparticles decreased cellular uptake by bone marrow derived monocytes by five times compared to rigid nanoparticles. They also found that particles with a bending stiffness higher than the cells were less efficient in their internalization. Similar studies further supported that nanoparticle elasticity governs nanoparticle uptake by macrophages via unique interactions between cell membranes and nanoparticles [89]. The stiffness and softening of nanoparticles determine ligand–receptor interactions and cell membrane wrapping, which, in turn, modulate their cellular binding and internalization rates.

### 3.2. Nanoparticle Active Targeting Immune Cells

Decorating nanoparticles with ligands that bind to neutrophil and macrophage surface receptors can improve the selectivity of nanoparticles. Although no active targeted nanoparticles are clinically available, numerous preclinical studies have demonstrated a better affinity and selectivity of active targeted nanoparticles than their passive counterparts. There is a broad spectrum of targeting ligands being investigated for neutrophil and macrophage targeted delivery.

Activated neutrophils change their surface receptor expression levels. Nanoparticle targeting to these receptors may be a novel strategy to specifically target neutrophils. CD11b belongs to a family of integrins and is highly expressed on activated neutrophils [18]. The anti-CD11b antibody can be employed as a high affinity ligand for nanoparticle targeting to activated neutrophils. Anti-CD11b coated nanoparticles increased neutrophil uptake in vivo by 10 times compared to non-targeting nanoparticles [90]. The Fcγ receptor is another receptor highly expressed on adherent neutrophils during inflammation and vascular diseases [91]. The binding of Fcγ receptors with IgG-opsonized particles and denatured protein nanoparticles mediated the neutrophil endocytosis of nanoparticles [92] for specifically delivering therapeutics adherent neutrophils [93].

There are more ligands of active cellular targeting for macrophages than neutrophils, as macrophages contain many scavenger receptors involved in receptor-mediated endocytosis. CD163 is one of the cysteine-rich scavenger receptors expressed on M2-like macrophages and plays a role in the resolution of inflammation [94]. Anti-CD163-linked nanoparticles were utilized to target CD163-overexpressed TAM [95]. CD206, a mannose receptor, is a C-type lectin presenting on the surface of macrophages. It plays a vital role in the recognition of pathogens via its pattern recognition domain [96]. Mannosylated nanoparticles have been commonly used for the active targeting of macrophages and have been shown to enhance their cellular uptake [97]. Other receptors and proteins expressed on macrophages include a folate receptor, a glucan receptor and legumain. Nanoparticles modified with folic acid [98], glucan [99] and alanine–alanine–asparagine peptides [100] were widely employed for macrophage targeting in experimental models and have enhanced therapeutic efficacy.

Precise targeting to neutrophils and macrophages is still challenging. The off-target effects may lead to systemic toxicity. Therefore, understanding the interactions between immune cells and nanoparticles and identifying biomarkers of subpopulations in immune cells are important in designing nanoparticles for clinical translation.

## 4. Nanoparticles for Anti-Inflammation Therapy

There are two essential objectives in anti-inflammation therapy: (1) suppressing overactive inflammatory responses to avoid self-damage; (2) restoring cellular functions to homeostasis. For these purposes, nanoparticles are designed to specifically target activated neutrophils or macrophages to control their activation and tissue infiltration for inflammation resolution. Nanoparticles also regulate the phenotype transition and secretion of immune cells. We will discuss several examples to illustrate immune regulations using nanoparticles.

### 4.1. Regulating Immune Cell Transmigration

Neutrophil tissue infiltration is a major source that drives inflammatory responses. If this movement is out of control, infiltrated neutrophils induce tissue damage. Therefore, regulating neutrophil recruitment may be a novel strategy to treat inflammatory diseases. However, drugs can not specifically target activated neutrophils in situ. Wang and colleagues [93] employed albumin nanoparticles to target activated neutrophils in the bloodstream and delivered piceatannol, a small molecule that blocks “outside-in” β_2_ integrin signaling pathways in leukocytes. The studies further showed that albumin nanoparticle uptake by neutrophils adherent to inflammatory endothelium was dependent on Fcγ receptors highly expressed on activated neutrophils. This approach effectively detached adherent neutrophils from the endothelium compared to free piceatannol, thus preventing neutrophil infiltration. Similarly, Tang et al. [101] was inspired by the platelet–neutrophil interaction in the inflammation response to generate platelet membrane-coated superparamagnetic iron oxide nanoparticles that can target activated neutrophils. They delivered piceatannol using nanoparticles to acute ischemic stroke tissues. The platelet membrane coated nanoparticles selectively targeted to activated neutrophils via the binding of P-selectin on platelets to P-selectin glycoprotein ligand-1 (PSGL-1) on activated neutrophils. This approach decreased neutrophil infiltration, thus preventing neuroinflammation. Interestingly, targeting to the inflammatory endothelium to block neutrophil–endothelium interactions is an alternative strategy to inhibit neutrophil infiltration [102].

Targeting signaling pathways of recruitment of monocytes/macrophages is another robust approach for anti-inflammation therapies. The chemokine C-C motif receptor 2 (CCR2) is a regulator for chemotaxis in monocytes and their release from bone marrow and spleen. A lipid-based nanoparticle has been used to deliver siRNA to silence CCR2 [103]. These studies showed that nanoparticles mainly accumulated in spleen, and selectively inhibited the migration of inflammatory monocytes rather than noninflammatory monocytes. This strategy showed the benefit to a wide range of diseases including cardiovascular diseases, cancer, and transplant rejection.

### 4.2. Depleting Immune Cells Using Nanoparticles

During inflammation, granulopoiesis and monocytopoiesis accelerate, resulting in an increase in the number of neutrophils and macrophages in circulation. An overwhelming number of immune cells has the potential to cause inflammatory disorders. Precisely regulating the numbers of neutrophils or macrophages is a new strategy to treat inflammatory disorders.

Apoptosis is a pathway to program cell death to maintain a constant number of neutrophils in bloodstream. This natural cell death is a molecular mechanism to preserve the immune homeostasis. However, inflammation responses rapidly increase the number of neutrophils in circulation, and the longevity results in vascular occlusion and neutrophil infiltration. Finally, it causes inflammatory diseases. Therefore, specifically targeting these activated neutrophils and controlling their numbers at the homeostatic level may be a novel strategy to treat inflammatory diseases.

**Figure 2:**
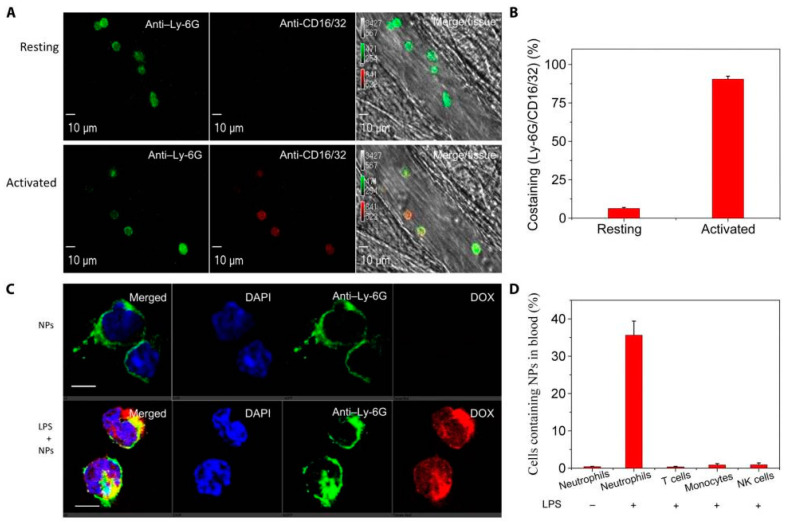
In situ nanoparticle targeting to activated neutrophils via Fcγ receptors. (**A**) Intravital microscopy of mouse cremaster muscle venules shows that neutrophil activation up-regulates Fcγ receptors. The resting neutrophils was established by no intrascrotal injection of TNF-a (0.5 mg per mouse) and the tail vein injection of Fcγ antibodies 3 h before performing intravital microscopy (top). Intrascrotal injection of TNF-a (0.5 mg per mouse) activated neutrophils. Alexa Fluor 647-labeled anti-mouse CD16/32 (red) and Alexa Fluor 488-labeled anti-mouse LY-6G (green) antibodies were intravenously injected to stain Fcγ receptors and neutrophils, respectively (bottom). Scale bars, 10 mm. (**B**) Percentages of co-staining between anti-mouse CD16/32 and anti-mouse LY-6G based on intravital images of (**A**). (**C**) Confocal laser scanning microscopy (CLSM) images of blood neutrophils from healthy mice or lipopolysaccharide (LPS)-challenged mice. Four hours after intraperitoneal LPS injection, doxorubicin conjugated bovine serum albumin nanoparticle (DOX-hyd-BSA) NPs were intravenously administered to a mouse. 3 h later, the mouse blood was collected, and neutrophils were isolated using anti-mouse LY-6G beads. Alexa Fluor 488-labeled antimouse LY- 6G antibody was used to label neutrophils. Scale bars, 10 mm. (**D**) Uptake of BSA NPs by blood leukocytes analyzed by flow cytometry. Neutrophils, T cells, monocytes, and natural killer (NK) cells were isolated from blood and stained by Alexa Fluor 647-labeled anti-mouse LY-6G, CD3, CD115, and CD335 antibodies, respectively. All data are expressed as means ± SD (three mice per group). (Reproduced from [105]; Published by American Association for the Advancement of Science (AAAS), 2019).

In one study, (R)-roscovitine delivery by polymersomes to neutrophils was dependent on scavenger receptors [104]. The polymersomes subsequently escaped the early endosomes through a pH-triggering disassembly of polymersomes that allowed delivery of R-roscovitine into the neutrophil cytosol without causing cellular activation. The R-roscovitine promoted neutrophil apoptosis leading to resolution of inflammation. Wang’s group developed a doxorubicin (DOX) conjugated bovine serum albumin nanoparticle (DOX-hyd-BSA) which selectively delivered DOX into activated neutrophils (Figure 2) [105]. This nanoparticle formulation induced neutrophil apoptosis and prevented inflammation responses. Notably, this approach increased mouse survival in sepsis and prevented brain damage in cerebral ischemia/reperfusion without suppressing systemic immunity.

Clodronate liposomes are the most commonly used formulations for macrophage depletion, developed by van Rooijen and colleagues early in 1990s [106]. Because of the nature of liposomes phagocytized by macrophages, clodronate liposomes can specifically deplete macrophages via an ATP-dependent mechanism. Clodronate liposomes have demonstrated effectiveness in depleting macrophages in inflammatory diseases of animal models [107,108,109,110]. However, complete removal of macrophages in the immune system may increase the risk of infections and dysfunction to systemic immunity [111]. Local administration of drugs may be a strategy to eliminate macrophages in certain tissues/organs, thus minimizing systemic toxicity. Doxorubicin (Dox) conjugated with CdSe/CdS/ZnS quantum dots was directly delivered to the lung via inhalation. Dox induced the apoptosis of alveolar macrophages and significantly prevented lung inflammatory injury [112]. Similarly, methotrexate (MTX)-loaded nanomicelles made of polycaprolactone-polyethylene glycol-polycaprolactone (PCL-PEG-PCL) triblock copolymer, formed a transdermal hydrogel and showed improved pharmacokinetics in treating rheumatoid arthritis. This formulation reduced hepatotoxicity and immunotoxicity [113].

Local administration of drugs is not feasible for most inflammatory diseases, such as cardiovascular and metabolic disorders, brain diseases and cancer, because drugs lack tissue penetration abilities after systemic delivery. However, systemic delivery of drugs requires delivery systems to have tissue specificity. Tang and his colleagues loaded simvastatin to high-density lipoprotein (S-HDL) nanoparticles and the nanoparticles suppressed atherosclerotic plaques in apolipoprotein E–deficient mice [114]. They found that S-HDL did not change the properties of macrophage recruitment, but interestingly observed that S-HDL inhibited the proliferation of macrophages in atherosclerotic plaques to control the inflammation response. In addition, targeting macrophages via their surface receptors is another strategy to manipulate macrophage functions. For instance, anti-PD-L1-conjuated lipid nanoparticles showed enhanced uptake by PD-L1 overexpressed TAMs and the nanoparticles loaded with the small-molecule CDK5 inhibitor, dinaciclib, dramatically killed TAMs in tumor microenvironments [115]. Nanoparticles were conjugated with mannose and protected with an extracellular pH-sensitive agent. When nanoparticles were at acidic tumor microenvironments, mannose was exposed to increase the uptake of TAMs. Using these nanoparticles, TAMs can be specifically depleted [116].

### 4.3. Controlling Cellular Phenotypes

Neutrophils are heterogeneous and highly plastic. The concept of neutrophil phenotypes has not been recognized for a long time since they are terminal cells and have a short lifespan [18]. However, comprehensive studies have discovered granulocytic myeloid-derived suppressor cells (G-MDSCs) in tumor microenvironments [117], and there were two phenotypes with anti-inflammatory or immunosuppressive features. They are called N1 for pro-inflammatory neutrophils and N2 for anti-inflammatory neutrophils [118]. It is still a controversial topic how to classify neutrophil populations. The role of these cells in inflammatory disease is still unclear. MDSCs play a double-edged role in inflammation-mediated diseases. MDSCs appeared to exacerbate [119] or to prevent [120,121] disease progression in autoimmune disorders.

Evidence supports that the phenotypes of neutrophils are dependent on the disease progress [118,122,123,124,125,126]. This property may provide the possibility to control neutrophil phenotypes for the treatment of inflammatory diseases. Current studies in this area are limited, and it may be useful to apply nanotechnologies to reprogram neutrophils to treat cancer and inflammatory diseases. In the future, new methods capable of determining biomarkers of subpopulations of neutrophils for their classification in healthy or pathologic conditions need to be developed [26,127,128,129], and these analyses will enable the rational design of targeted therapies.

Phenotypes of macrophages have been extensively studied. Specifically, pro-inflammatory mediators, such as LPS, IFN-γ and TNF, toll-like receptor 4 (TLR4), drive macrophages to transform to the M1 phenotype. A novel class of hexapeptides-gold nanoparticle hybrids were recently reported to inhibit TLR signaling pathways in macrophages [130,131,132]. The authors found that hydrophobicity and aromatic ring structures of amino acids in the peptides were essential for modulating TLR4 responses. This approach may tune the inhibition of TLR4. Another study revealed that nanohybrids induced the polarization of alveolar M2 macrophages, but reduced M1 macrophages in an acute lung injury model [133].

Transformation of M2 phenotypes can be stimulated by interleukin 4 and 13 (IL-4 and IL-13), therefore, IL-4 or IL-13 may be therapeutics for anti-inflammation and tissue repair. Raimondo and Mooney [134] injected gold nanoparticles conjugated with IL-4 into injured murine skeletal muscles and found that the muscles were repaired and the muscle force increased by 40% compared to the treatment with vehicles. The therapeutic effect is associated with the polarization of M2 macrophages. This feature was not observed in the treatments with soluble IL-4 and depletion of macrophages, suggesting the importance of phenotypic regulation for tissue repair.

Studies have also shown that nanoparticles can regulate genetic expression [135] and intracellular calcium [136] to manipulate the polarization of macrophages for anti-inflammation. Priming M2 macrophages is a novel method to increase their efferocytosis to initiate the resolution phase of inflammation. In a previous study, nanoparticles were comprised of a poly(lactide-co-glycolide) as a core and a coating with phosphatidylserine (PS)-supplemented cell plasma membrane (PS-MNP) to mimic the apoptotic cell surface [137]. The macrophage engulfment of these nanoparticles elicited the polarization of M2 macrophages, and subsequently, inflammation was reduced via the NF-κB pathway. It should be noted that transition to the anti-inflammatory phenotype (M2) is not universal for anti-inflammation. For example, in a severe infection, nanoparticles that promoted the activation of M1, but inhibited the M2 phenotype showed an anti-infection benefit [138]. This dilemma between M1 and M2 in anti-inflammation requires further investigation.

### 4.4. Regulating Cellular Functions

NETosis is a form of neutrophil death characterized by the release of decondensed chromatin and granular contents to the extracellular space. Specifically, NETosis is initiated by activation of NADPH oxidase that produces reactive oxygen species (ROS). ROS activates protein-arginine deiminase 4 (PAD4) to convert arginine to citrulline on histones, which induces chromatin decondensation [139,140,141]. NETosis contributes to acute and chronic inflammatory disorders [141,142]. Targeting the NETosis pathways may be a novel strategy to block NETs-mediated pathologies [52,143,144]. Nanoparticle-based delivery systems offer tools to target NETosis for improved therapies. Sivelestat, an inhibitor of NETs, delivered by inter-bilayer-crosslinked multilamellar vesicles (ICMVs) rescued mice from the LPS-induced endotoxic shock [145]. Another study showed that nanoparticles coated with murine siglec-E ligand, α 2,8-linked sialic acid residues, can target Siglec receptors, thus decreasing the inflammation response [146]. Interestingly, an α 2,8-sialylated nanoparticle inhibited ROS in neutrophils, thus decreasing the formation of NETs [147]. Although NETs are complex and the molecular mechanisms are not fully understood, current studies have shown promise for targeting NETs for anti-inflammation therapies.

Tumor necrosis factor α (TNF-α) is a potent cytokine that initiates an inflammatory cascade and severe tissue damage. Targeting macrophages using nanoparticles to silence TNF-α is widely reported to reduce inflammation in rheumatoid arthritis (RA), intestinal inflammation/injury and LPS-induced inflammation. Nanoparticles are usually coated with ligands that target macrophages and nanoparticles incorporate siRNA that silences the TNF-α gene. These formulations can inhibit the inflammation response in macrophages [148,149,150,151]. Some nanoparticles have been designed for oral delivery of siRNA to silence TNF-α via targeting intestinal macrophages [152,153,154].

Macrophages also play a major role in the pathogenesis of obesity, atherosclerosis and diabetes. For example, Luo et al. [155] developed cationic lipid-assisted PEG-b-PLGA nanoparticles (CLAN) loaded with a plasmid encoding a guide RNA of Ntn1 (sgNtn1), thus specifically manipulating macrophages. Using this approach, nanoparticles improved the therapy of type 2 diabetes in a mouse model.

## 5. Restoring Immune Functions Using Nanoparticles

Neutrophils and macrophages are immune sentinel cells that attack pathogens and repair damage to the host. It is important to regulate their functions for specific purposes.

### 5.1. Restoring Neutrophil Functions

Cancer is strongly associated with immunosuppressive environments because there are a large number of infiltrated neutrophils. These neutrophils lose their ability to kill tumor cells instead of promoting tumor progress. They are called tumor-associated neutrophils (TANs). Intense studies have shown that TANs are regulated by the IL-23–IL-17 axis and CXCR2 [156,157,158,159], therefore they are potential targets for cancer immunotherapies. Although targeting of TANs has been proposed to treat cancer [160], it is challenging to specifically target them due to blood vessel barriers and cell specificity [161,162,163].

Severe infections and sepsis derail normal neutrophil functions. For example, aging causes defects in neutrophils’ abilities as a host defender, so neutrophils lose ability to clear pathogens. Most importantly, the role of “bystanders” caused more tissue damage in elderly individuals [164]. Another interesting study showed that targeting CXCR2 pathways in neutrophils increased mouse survival from sepsis because the IL-33 cytokine restored neutrophil recruitment, clearing bacteria [165]. In a recent study, PLGA nanoparticles were employed to deliver curcumin. Curcumin is a sarcoplasmic/endoplasmic reticulum calcium pump (SERCA) inhibitor that induces endoplasmic reticulum (ER) calcium release [166]. The results showed that nanoparticle formulations restored neutrophils ability to generate intracellular ROS that reactivated the neutrophil’s antibacterial ability to treat a chronic granulomatous disease.

### 5.2. Reactivating Macrophage Immunity

Tumor-associated macrophages (TAMs) play a key role in tumor resistance, so targeting TAMS is a novel strategy for tumor immunotherapy [167]. The axes of CD47-signal regulatory protein α (SIRPα) and macrophage colony stimulating factor (MCSF)-colony stimulating factor 1 receptor (CSF1-R) between cancer cells and macrophages are important in inhibiting tumor killing by TAMs. Ramesh and colleagues developed a lipid-based nanoparticle loaded with two inhibitors to target TAMs [168]. They found that nanoparticles activated macrophages and enhanced their phagocytosis. To specifically target M2 macrophages, they conjugated antibodies (CD206, a marker of M2) to nanoparticles. The results showed that nanoparticles repolarized macrophages and dramatically decreased tumor growth.

**Figure 3:**
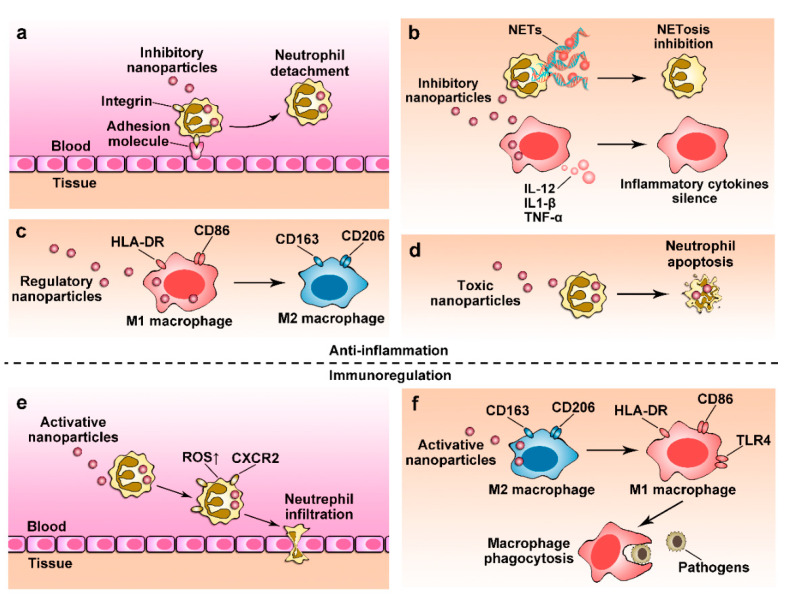
Targeting neutrophils and macrophages using nanoparticles for immunotherapies in inflammatory disorders. (**a**) Albumin nanoparticles loaded with piceatannol inhibit the neutrophil adhesion to endothelium, thus blocking neutrophil infiltration [93]. (**b**) Nanoparticles inhibit the functions of activated neutrophils and macrophages to reduce pro-inflammatory factors [149] and NETs release [145]. (**c**) Nanoparticles transform macrophages from the pro-inflammatory M1 phenotype (biomarkers of human leukocyte antigen-DR isotype (HLA-DR) and CD86) to anti-inflammatory M2 phenotype (biomarkers of CD163 and CD206) [133]. (**d**) DOX-loaded Nanoparticles induce neutrophil apoptosis to induce the resolution of inflammation [105]. (**e**) Nanoparticles activate neutrophil by up-regulating intracellular reactive oxygen species (ROS) level and CXCR2 to restore neutrophil pathogen clearance [166]. (**f**) Nanoparticles induce the transition of immunosuppressive M2 macrophages to immunoenhancement M1 macrophages to enhance phagocytosis [168].

Intracellular infections in macrophages are difficult to treat because pathogens impair macrophage functions. Targeting infected macrophages using nanoparticles is proposed to eliminate intracellular pathogens. Bose and colleagues employed apoptotic bodies (ReApoBds) derived from cancer cells as “nano decoys” to deliver vancomycin into macrophages, highlighting its effective intracellular bacteria killing ability [169]. Figure 3 shows the current approaches and technologies used to target neutrophils and macrophages using nanoparticles to improve the therapies of inflammatory diseases.

## 6. Delivery of Nanotherapeutics via Leukocytes

Cell-mediated delivery of therapeutics has emerged as a great potential drug delivery tool because cells possess tissue targeting mechanisms and overcome biological barriers that are faced by synthetic nanoparticle drug delivery systems [90,161]. There are two methods: (1) hijacking leukocytes in vivo to deliver nanotherapeutics; (2) loading nanoparticles to leukocytes in vitro, then infusing the leukocytes back to animals. We will discuss each approach and their advantages and disadvantages.

### 6.1. In Situ Hijacking of Leukocytes to Deliver Nanotherapeutics

Neutrophils and macrophages have the ability to spontaneously migrate to inflamed tissues, therefore, their tropism allows for the delivery of therapeutics to injured tissues if nanoparticles specifically bind to neutrophils or macrophages in situ. This approach allows us to escape the complicated cell isolation and in vitro cellular loading of nanotherapeutics, but requires the nanoparticles to specifically interact with the targeted leukocytes in vivo.

Chu et al. for the first time reported that neutrophils transported bovine serum albumin (BSA) nanoparticles across blood vessel barriers after neutrophils specifically internalized nanoparticles in vivo [170]. BSA nanoparticles loaded with an NF-κB inhibitor, (2-([aminocarbonyl]-amino)-5-(4-fluorophenyl)-3-thiophenecarboxamide) (TPCA-1) or an antibiotic, cefoperazone acid (Cefo-A), enhanced drug delivery in the lung, thus alleviating acute lung inflammation/injury and lung infection by *P. aeruginosa*, respectively. In addition, Zhang and colleagues showed that tripeptide Pro-Gly-Pro (PGP)-modified poly-L-lysine (DGL) nanoparticles delivered catalase (CAT) to the brain for therapy of ischemia stroke [171]. In the study, they showed that neutrophils took the nanoparticles through binding of PGP to the CXCR2 receptor, and neutrophils transported nanoparticles to inflammatory sites of ischemia stroke.

Monocytes/macrophages have a low percentage of leukocytes, therefore, targeting monocytes/macrophages may not be an efficient method to deliver nanotherapeutics. A study showed that cyclic arginine-glycine-aspartic (cRGD) peptide-modified liposomes delivered a neuroprotective agent, edaravone (ER) to the brain through the transmigration of neutrophils and monocytes. [172].

Drug release from nanoparticle-laden leukocytes after they arrive at targeting sites is complicated. Several studies have suggested nanoparticle release via intercellular transport [172] and cell death [173,174]. Successful delivery of nanotherapeutics via leukocytes is important to maintain cell movement ability before they arrive at targeting sites, but precise investigation into this question is needed in the future.

### 6.2. Nanoparticle-Laden Leukocytes In Vitro

To increase drug loading in leukocytes and maintain cellular functions, nanoparticles are used to protect drug release in leukocytes. Studies have used liposomes that were co-incubated with neutrophils in order to obtain nanoparticle-laden cells, and the cells were intravenously administered to animals for treatment of atherosclerosis [175] and rheumatoid arthritis [176]. These studies lacked rigorous investigation into whether nanoparticle-laden leukocytes maintain cellular movements to targeting sites and whether they compete endogenous leukocytes [162,163].

Decoration of nanoparticles on the cell surface without influencing cell functions is a new approach to develop cell-based therapeutics. Specifically, a cell backpack has been designed to attach nanoparticles onto the macrophage surface but prevents their internalization. These cell backpacks are made from multilayer polymer nanoparticles that can have cell adhesion, drug loading and controlled release properties. The backpacks have exhibited significant prolonged retention after they were adherent to the macrophage surface without internalization compared to spherical counterparts [177]. Interestingly, the backpacks on monocytes/macrophages successfully delivered anti-inflammatory agents to inflamed organs [178,179]. In addition, Shields and colleagues developed a backpack that robustly adhered to the macrophage surface for several days and regulated cellular phenotypes in vivo [180]. In the study, they incorporated IFN-γ in a polyvinyl alcohol (PVA) layer between PLGA and further attached these prepared backpacks onto macrophages through a cell-adhesive layer. Sustained release of IFN-γ promoted the M1 macrophage phenotype, leading to a reduction in metastasis and tumor growth.

## 7. Immunotoxicity in Leukocytes of Nanoparticle Uptake

Current studies are focused on how nanoparticle-based therapeutics target leukocytes and regulate leukocyte functions for improved immunotherapies in a wide range of diseases. However, investigations into whether nanoparticles themselves initiate inflammatory responses are lacking. Neutrophils and macrophages express high levels of pattern recognition receptors (PRRs). If nanoparticles bind to PRRs, they may trigger inflammatory responses. When nanoparticles circulate in the bloodstream, they are quickly coated with proteins. The corona proteins on nanoparticles act as nanoparticle-associated molecular patterns (NAMPs) to initiate pro-inflammatory responses [17,181,182]. One study has indicated that nanoparticles with protein mimics [183] bound to toll-like receptors (TLRs) result in inflammatory responses [184,185]. Although some studies have claimed that there is less of an influence of nanoparticle uptake on the functions of neutrophils [90,186] and macrophages [187], the experimental conditions (such as nanoparticle concentrations and material compositions) may be critical. Therefore, further investigation in the future is needed.

NETosis is a hall marker of neutrophil activation and has the potential to cause immunotoxicity. It has been found that nanoparticle sizes and shapes are critical factors for formation of NETs [141,188]. As an example, small-size nanoparticles (10–40 nm) quickly damaged (<20 min) plasma membranes and the lysosomal compartment, leading to the formation of NETs, whereas particles sized with 100–1000 nm did not cause NETs [189].

The material properties of nanoparticles may be effective for neutrophil functioning. Catherine et al. [64] found that drug-free carboxylate-modified particles were rapidly sequestered by neutrophils within the bloodstream and hindered neutrophil adhesion to the inflamed mesentery vascular wall. In a model of acute lung injury, these nanoparticles reduced neutrophil accumulation in the airways, diverting neutrophils to the liver. There are two possible explanations for this phenomenon: (1) the nanoparticles induced neutrophil apoptosis and recalled them back to recycling organs; (2) the nanoparticle induced local inflammation in liver that redistributed neutrophils. It is not clear what molecular mechanism regulates the neutrophil uptake of nanoparticles. This nonspecific neutrophil binding to nanoparticles may be associated with concentrations of administered nanoparticles, and further investigation is needed. Inflammation was decreased using poly(DL-lactide-co-glycolide) (PLG) nanoparticles [190,191], but similar nanoparticles formed from 50:50 PLG with low molecular weight (PLG-L), high molecular weight (PLG-H) and poly(DL-lactide) (PDLA) showed inefficiencies in ameliorating inflammatory responses [192]. The mechanism is not fully understood.

Similar studies have been performed in macrophages. A recent study revealed that uptake of gold nanoparticles was strongly dependent on macrophage phenotypes. The results demonstrated that “regulatory” M2 phenotype macrophages expressing higher CD163 markers took up more nanoparticles. Interestingly, the nanoparticle uptake did not alter macrophage differentiation, death or phagocytosis, but significantly inhibited the secretion of TNF-α in response to inflammatory challenge [193].

Nanoparticle-induced immunotoxicity includes two aspects: (1) immunostimulation-caused damage, such as a cytokine storm and lymphocyte activation; (2) immunosuppression-induced infections or tumorigenesis. Understanding the undesired immune responses and lymphocyte behavior associated with nanoparticles is crucial to reduce immunotoxicity and facilitate the clinical transformation of nanoparticles. The topic on how nanoparticles themselves regulate leukocyte functions, such as activation and loss of their function, is a new research area, and this study is important for the development of nanotherapeutics to treat inflammatory diseases.

## 8. Conclusions

Here, we have reviewed the current status of the targeting of leukocytes (neutrophils and macrophages) using nanoparticles for developing nanotherapeutics to treat inflammatory diseases. The studies mentioned here show that nanoparticles can deliver drugs into neutrophils or macrophages to inhibit their activation and inflammation responses or to restore their functions for boosting their immunity. To treat excessive inflammation-associated diseases, such as lung inflammation/injury and stroke, delivery of anti-inflammatory agents is required. However, for bacterial infections, increasing the defense of leukocytes is required. Another area is to hijack leukocyte transmigration to deliver nanotherapeutics across blood vessel barriers. This is a new research area, and the success of this novel concept has the potential to address fundamental questions in nanomedicine to treat cancer and other vascular diseases [161] Finally, nanoparticle-induced immunotoxicity should be taken into consideration before further application.

Although there are many studies in which nanoparticles have been shown to interact with neutrophils or macrophages in vivo, the binding and uptake of nanoparticles have not been fully addressed. Development of novel imaging technologies (such as intravital microscopy) and rationally designed nanoparticles to specifically target leukocytes in vivo are needed [93,194]. Specificity of nanoparticles to target neutrophils or macrophages in vivo is challenging because neutrophils and macrophages share similar biomarkers with other adaptive immune cells. Understanding of immunology and collaborations between biologists and clinicians are needed to develop interdisciplinary research.

## References

[1] Medzhitov R. (2008). Origin and physiological roles of inflammation. Nature.

[2] Henson P.M. (2005). Dampening inflammation. Nat. Immunol..

[3] Gao J., Wang S., Dong X., Leanse L.G., Dai T., Wang Z. (2020). Co-delivery of resolvin d1 and antibiotics with nanovesicles to lungs resolves inflammation and clears bacteria in mice. Commun. Biol..

[4] Dong X., Gao J., Su Y., Wang Z. (2020). Nanomedicine for ischemic stroke. Int. J. Mol. Sci..

[5] Furman D., Campisi J., Verdin E., Carrera-Bastos P., Targ S., Franceschi C., Ferrucci L., Gilroy D.W., Fasano A., Miller G.W. (2019). Chronic inflammation in the etiology of disease across the life span. Nat. Med..

[6] Amulic B., Cazalet C., Hayes G.L., Metzler K.D., Zychlinsky A. (2012). Neutrophil function: From mechanisms to disease. Annu. Rev. Immunol..

[7] Summers C., Rankin S.M., Condliffe A.M., Singh N., Peters A.M., Chilvers E.R. (2010). Neutrophil kinetics in health and disease. Trends Immunol..

[8] Wright H.L., Moots R.J., Bucknall R.C., Edwards S.W. (2010). Neutrophil function in inflammation and inflammatory diseases. Rheumatology.

[9] Lee S., Huen S., Nishio H., Nishio S., Lee H.K., Choi B.S., Ruhrberg C., Cantley L.G. (2011). Distinct macrophage phenotypes contribute to kidney injury and repair. J. Am. Soc. Nephrol. JASN.

[10] Serhan C.N., Savill J. (2005). Resolution of inflammation: The beginning programs the end. Nat. Immunol..

[11] Soehnlein O., Steffens S., Hidalgo A., Weber C. (2017). Neutrophils as protagonists and targets in chronic inflammation. Nat. Rev. Immunol..

[12] Grieshaber-Bouyer R., Nigrovic P.A. (2019). Neutrophil heterogeneity as therapeutic opportunity in immune-mediated disease. Front. Immunol..

[13] Lee C.H., Choi E.Y. (2018). Macrophages and inflammation. J. Rheum. Dis..

[14] Mejbri M., Theodoropoulou K., Hofer M., Cimaz R. (2020). Interleukin-1 blockade in systemic juvenile idiopathic arthritis. Paediatr. Drugs.

[15] De Benedetti F., Brunner H.I., Ruperto N., Kenwright A., Wright S., Calvo I., Cuttica R., Ravelli A., Schneider R., Woo P. (2012). Randomized trial of tocilizumab in systemic juvenile idiopathic arthritis. N. Engl. J. Med..

[16] Boraschi D., Italiani P., Palomba R., Decuzzi P., Duschl A., Fadeel B., Moghimi S.M. (2017). Nanoparticles and innate immunity: New perspectives on host defence. Semin. Immunol..

[17] Fadeel B. (2012). Clear and present danger? Engineered nanoparticles and the immune system. Swiss Med Wkly..

[18] Kolaczkowska E., Kubes P. (2013). Neutrophil recruitment and function in health and inflammation. Nat. Rev. Immunol..

[19] Sheshachalam A., Srivastava N., Mitchell T., Lacy P., Eitzen G. (2014). Granule protein processing and regulated secretion in neutrophils. Front. Immunol..

[20] Brinkmann V., Reichard U., Goosmann C., Fauler B., Uhlemann Y., Weiss D.S., Weinrauch Y., Zychlinsky A. (2004). Neutrophil extracellular traps kill bacteria. Science.

[21] Gomez Perdiguero E., Klapproth K., Schulz C., Busch K., Azzoni E., Crozet L., Garner H., Trouillet C., de Bruijn M.F., Geissmann F. (2015). Tissue-resident macrophages originate from yolk-sac-derived erythro-myeloid progenitors. Nature.

[22] Epelman S., Lavine K.J., Randolph G.J. (2014). Origin and functions of tissue macrophages. Immunity.

[23] Mowat A.M., Scott C.L., Bain C.C. (2017). Barrier-tissue macrophages: Functional adaptation to environmental challenges. Nat. Med..

[24] Sica A., Erreni M., Allavena P., Porta C. (2015). Macrophage polarization in pathology. Cell. Mol. Life Sci. CMLS.

[25] Parisi L., Gini E., Baci D., Tremolati M., Fanuli M., Bassani B., Farronato G., Bruno A., Mortara L. (2018). Macrophage polarization in chronic inflammatory diseases: Killers or builders?. J. Immunol. Res..

[26] Adrover J.M., Nicolas-Avila J.A., Hidalgo A. (2016). Aging: A temporal dimension for neutrophils. Trends Immunol..

[27] Oishi Y., Manabe I. (2016). Macrophages in age-related chronic inflammatory diseases. NPJ Aging Mech. Dis..

[28] Nemeth T., Sperandio M., Mocsai A. (2020). Neutrophils as emerging therapeutic targets. Nat. Rev. Drug Discov..

[29] Chawla A., Nguyen K.D., Goh Y.P. (2011). Macrophage-mediated inflammation in metabolic disease. Nat. Rev. Immunol..

[30] Zhang C.Y., Gao J., Wang Z. (2018). Bioresponsive nanoparticles targeted to infectious microenvironments for sepsis management. Adv. Mater..

[31] Hotchkiss R.S., Monneret G., Payen D. (2013). Sepsis-induced immunosuppression: From cellular dysfunctions to immunotherapy. Nat. Rev. Immunol..

[32] Sonego F., Castanheira F.V., Ferreira R.G., Kanashiro A., Leite C.A., Nascimento D.C., Colon D.F., Borges Vde F., Alves-Filho J.C., Cunha F.Q. (2016). Paradoxical roles of the neutrophil in sepsis: Protective and deleterious. Front. Immunol..

[33] Sonego F., Alves-Filho J.C., Cunha F.Q. (2014). Targeting neutrophils in sepsis. Expert Rev. Clin. Immunol..

[34] Porta C., Rimoldi M., Raes G., Brys L., Ghezzi P., Di Liberto D., Dieli F., Ghisletti S., Natoli G., De Baetselier P. (2009). Tolerance and m2 (alternative) macrophage polarization are related processes orchestrated by p50 nuclear factor kappab. Proc. Natl. Acad. Sci. USA.

[35] Mizuma A., Yenari M.A. (2017). Anti-inflammatory targets for the treatment of reperfusion injury in stroke. Front. Neurol..

[36] Garcia-Prieto J., Villena-Gutierrez R., Gomez M., Bernardo E., Pun-Garcia A., Garcia-Lunar I., Crainiciuc G., Fernandez-Jimenez R., Sreeramkumar V., Bourio-Martinez R. (2017). Neutrophil stunning by metoprolol reduces infarct size. Nat. Commun..

[37] Anzai A., Choi J.L., He S., Fenn A.M., Nairz M., Rattik S., McAlpine C.S., Mindur J.E., Chan C.T., Iwamoto Y. (2017). The infarcted myocardium solicits gm-csf for the detrimental oversupply of inflammatory leukocytes. J. Exp. Med..

[38] Banati R.B., Gehrmann J., Schubert P., Kreutzberg G.W. (1993). Cytotoxicity of microglia. Glia.

[39] Barone F.C., Arvin B., White R.F., Miller A., Webb C.L., Willette R.N., Lysko P.G., Feuerstein G.Z. (1997). Tumor necrosis factor-α. A mediator of focal ischemic brain injury. Stroke.

[40] Han D., Liu H., Gao Y. (2020). The role of peripheral monocytes and macrophages in ischemic stroke. Neurol. Sci..

[41] Mouton A.J., Li X., Hall M.E., Hall J.E. (2020). Obesity, hypertension, and cardiac dysfunction: Novel roles of immunometabolism in macrophage activation and inflammation. Circ. Res..

[42] Kammoun H.L., Kraakman M.J., Febbraio M.A. (2014). Adipose tissue inflammation in glucose metabolism. Rev. Endocr. Metab. Disord..

[43] Lackey D.E., Olefsky J.M. (2016). Regulation of metabolism by the innate immune system. Nat. Rev. Endocrinol..

[44] Elgazar-Carmon V., Rudich A., Hadad N., Levy R. (2008). Neutrophils transiently infiltrate intra-abdominal fat early in the course of high-fat feeding. J. Lipid Res..

[45] Talukdar S., Oh D.Y., Bandyopadhyay G., Li D., Xu J., McNelis J., Lu M., Li P., Yan Q., Zhu Y. (2012). Neutrophils mediate insulin resistance in mice fed a high-fat diet through secreted elastase. Nat. Med..

[46] Barbarin V., Xing Z., Delos M., Lison D., Huaux F. (2005). Pulmonary overexpression of il-10 augments lung fibrosis and th2 responses induced by silica particles. Am. J. Physiol. Lung Cell. Mol. Physiol..

[47] Lee C.G., Homer R.J., Zhu Z., Lanone S., Wang X., Koteliansky V., Shipley J.M., Gotwals P., Noble P., Chen Q. (2001). Interleukin-13 induces tissue fibrosis by selectively stimulating and activating transforming growth factor β_1_. J. Exp. Med..

[48] Naura A.S., Zerfaoui M., Kim H., Abd Elmageed Z.Y., Rodriguez P.C., Hans C.P., Ju J., Errami Y., Park J., Ochoa A.C. (2010). Requirement for inducible nitric oxide synthase in chronic allergen exposure-induced pulmonary fibrosis but not inflammation. J. Immunol..

[49] Laval J., Ralhan A., Hartl D. (2016). Neutrophils in cystic fibrosis. Biol. Chem..

[50] Bazzan E., Turato G., Tine M., Radu C.M., Balestro E., Rigobello C., Biondini D., Schiavon M., Lunardi F., Baraldo S. (2017). Dual polarization of human alveolar macrophages progressively increases with smoking and copd severity. Respir. Res..

[51] Khandpur R., Carmona-Rivera C., Vivekanandan-Giri A., Gizinski A., Yalavarthi S., Knight J.S., Friday S., Li S., Patel R.M., Subramanian V. (2013). Nets are a source of citrullinated autoantigens and stimulate inflammatory responses in rheumatoid arthritis. Sci. Transl. Med..

[52] Lood C., Blanco L.P., Purmalek M.M., Carmona-Rivera C., De Ravin S.S., Smith C.K., Malech H.L., Ledbetter J.A., Elkon K.B., Kaplan M.J. (2016). Neutrophil extracellular traps enriched in oxidized mitochondrial DNA are interferogenic and contribute to lupus-like disease. Nat. Med..

[53] Fulop T., Larbi A., Douziech N., Fortin C., Guerard K.P., Lesur O., Khalil A., Dupuis G. (2004). Signal transduction and functional changes in neutrophils with aging. Aging Cell.

[54] Tseng C.W., Liu G.Y. (2014). Expanding roles of neutrophils in aging hosts. Curr. Opin. Immunol..

[55] Hearps A.C., Martin G.E., Angelovich T.A., Cheng W.J., Maisa A., Landay A.L., Jaworowski A., Crowe S.M. (2012). Aging is associated with chronic innate immune activation and dysregulation of monocyte phenotype and function. Aging Cell.

[56] Bonotis K., Krikki E., Holeva V., Aggouridaki C., Costa V., Baloyannis S. (2008). Systemic immune aberrations in alzheimer’s disease patients. J. Neuroimmunol..

[57] Fiala M., Lin J., Ringman J., Kermani-Arab V., Tsao G., Patel A., Lossinsky A.S., Graves M.C., Gustavson A., Sayre J. (2005). Ineffective phagocytosis of amyloid-beta by macrophages of alzheimer’s disease patients. J. Alzheimer’s Dis. JAD.

[58] Zenaro E., Pietronigro E., Della Bianca V., Piacentino G., Marongiu L., Budui S., Turano E., Rossi B., Angiari S., Dusi S. (2015). Neutrophils promote alzheimer’s disease-like pathology and cognitive decline via lfa-1 integrin. Nat. Med..

[59] Torchilin V.P. (2014). Multifunctional, stimuli-sensitive nanoparticulate systems for drug delivery. Nat. Rev. Drug Discov..

[60] Wang A.Z., Langer R., Farokhzad O.C. (2012). Nanoparticle delivery of cancer drugs. Annu. Rev. Med..

[61] Wang Z., Tiruppathi C., Cho J., Minshall R.D., Malik A.B. (2011). Delivery of nanoparticle: Complexed drugs across the vascular endothelial barrier via caveolae. IUBMB Life.

[62] Gao J., Dong X., Wang Z. (2020). Generation, purification and engineering of extracellular vesicles and their biomedical applications. Methods.

[63] Gao J., Chu D., Wang Z. (2016). Cell membrane-formed nanovesicles for disease-targeted delivery. J. Control Release.

[64] Fromen C.A., Kelley W.J., Fish M.B., Adili R., Noble J., Hoenerhoff M.J., Holinstat M., Eniola-Adefeso O. (2017). Neutrophil-particle interactions in blood circulation drive particle clearance and alter neutrophil responses in acute inflammation. ACS Nano.

[65] Moghimi S.M., Simberg D. (2017). Complement activation turnover on surfaces of nanoparticles. Nano Today.

[66] Vu V.P., Gifford G.B., Chen F., Benasutti H., Wang G., Groman E.V., Scheinman R., Saba L., Moghimi S.M., Simberg D. (2019). Immunoglobulin deposition on biomolecule corona determines complement opsonization efficiency of preclinical and clinical nanoparticles. Nat. Nanotechnol..

[67] Davies L.C., Jenkins S.J., Allen J.E., Taylor P.R. (2013). Tissue-resident macrophages. Nat. Immunol..

[68] Lapenna A., De Palma M., Lewis C.E. (2018). Perivascular macrophages in health and disease. Nat. Rev. Immunol..

[69] Baer C., Squadrito M.L., Iruela-Arispe M.L., De Palma M. (2013). Reciprocal interactions between endothelial cells and macrophages in angiogenic vascular niches. Exp. Cell Res..

[70] Lewis C.E., Harney A.S., Pollard J.W. (2016). The multifaceted role of perivascular macrophages in tumors. Cancer Cell.

[71] Mendes-Jorge L., Ramos D., Luppo M., Llombart C., Alexandre-Pires G., Nacher V., Melgarejo V., Correia M., Navarro M., Carretero A. (2009). Scavenger function of resident autofluorescent perivascular macrophages and their contribution to the maintenance of the blood-retinal barrier. Investig. Ophthalmol. Vis. Sci..

[72] Ferris S.T., Carrero J.A., Mohan J.F., Calderon B., Murphy K.M., Unanue E.R. (2014). A minor subset of batf3-dependent antigen-presenting cells in islets of langerhans is essential for the development of autoimmune diabetes. Immunity.

[73] Stamatiades E.G., Tremblay M.E., Bohm M., Crozet L., Bisht K., Kao D., Coelho C., Fan X., Yewdell W.T., Davidson A. (2016). Immune monitoring of trans-endothelial transport by kidney-resident macrophages. Cell.

[74] Niemi M., Sharpe R.M., Brown W.R. (1986). Macrophages in the interstitial tissue of the rat testis. Cell Tissue Res..

[75] Dai Q., Wilhelm S., Ding D., Syed A.M., Sindhwani S., Zhang Y., Chen Y.Y., MacMillan P., Chan W.C.W. (2018). Quantifying the ligand-coated nanoparticle delivery to cancer cells in solid tumors. ACS Nano.

[76] Bisso P.W., Gaglione S., Guimaraes P.P.G., Mitchell M.J., Langer R. (2018). Nanomaterial interactions with human neutrophils. ACS Biomater. Sci. Eng..

[77] Perry J.L., Reuter K.G., Luft J.C., Pecot C.V., Zamboni W., DeSimone J.M. (2017). Mediating passive tumor accumulation through particle size, tumor type, and location. Nano Lett..

[78] Tong X., Wang Z., Sun X., Song J., Jacobson O., Niu G., Kiesewetter D.O., Chen X. (2016). Size dependent kinetics of gold nanorods in epr mediated tumor delivery. Theranostics.

[79] Safari H., Kelley W.J., Saito E., Kaczorowski N., Carethers L., Shea L.D., Eniola-Adefeso O. (2020). Neutrophils preferentially phagocytose elongated particles-an opportunity for selective targeting in acute inflammatory diseases. Sci. Adv..

[80] Kelley W.J., Fromen C.A., Lopez-Cazares G., Eniola-Adefeso O. (2018). Pegylation of model drug carriers enhances phagocytosis by primary human neutrophils. Acta Biomater..

[81] Yu S.S., Lau C.M., Thomas S.N., Jerome W.G., Maron D.J., Dickerson J.H., Hubbell J.A., Giorgio T.D. (2012). Size- and charge-dependent non-specific uptake of pegylated nanoparticles by macrophages. Int. J. Nanomed..

[82] Champion J.A., Mitragotri S. (2006). Role of target geometry in phagocytosis. Proc. Natl. Acad. Sci. USA.

[83] Geng Y., Dalhaimer P., Cai S., Tsai R., Tewari M., Minko T., Discher D.E. (2007). Shape effects of filaments versus spherical particles in flow and drug delivery. Nat. Nanotechnol..

[84] Champion J.A., Walker A., Mitragotri S. (2008). Role of particle size in phagocytosis of polymeric microspheres. Pharm. Res..

[85] Saha K., Rahimi M., Yazdani M., Kim S.T., Moyano D.F., Hou S., Das R., Mout R., Rezaee F., Mahmoudi M. (2016). Regulation of macrophage recognition through the interplay of nanoparticle surface functionality and protein corona. ACS Nano.

[86] Ergen C., Heymann F., Al Rawashdeh W., Gremse F., Bartneck M., Panzer U., Pola R., Pechar M., Storm G., Mohr N. (2017). Targeting distinct myeloid cell populations in vivo using polymers, liposomes and microbubbles. Biomaterials.

[87] Zhang L.W., Monteiro-Riviere N.A. (2009). Mechanisms of quantum dot nanoparticle cellular uptake. Toxicol. Sci..

[88] Palomba R., Palange A.L., Rizzuti I.F., Ferreira M., Cervadoro A., Barbato M.G., Canale C., Decuzzi P. (2018). Modulating phagocytic cell sequestration by tailoring nanoconstruct softness. ACS Nano.

[89] Hui Y., Yi X., Wibowo D., Yang G., Middelberg A.P.J., Gao H., Zhao C.X. (2020). Nanoparticle elasticity regulates phagocytosis and cancer cell uptake. Sci. Adv..

[90] Chu D., Dong X., Zhao Q., Gu J., Wang Z. (2017). Photosensitization priming of tumor microenvironments improves delivery of nanotherapeutics via neutrophil infiltration. Adv. Mater..

[91] Nakatani K., Takeshita S., Tsujimoto H., Kawamura Y., Kawase H., Sekine I. (1999). Regulation of the expression of fc gamma receptor on circulating neutrophils and monocytes in kawasaki disease. Clin. Exp. Immunol..

[92] Chen K., Nishi H., Travers R., Tsuboi N., Martinod K., Wagner D.D., Stan R., Croce K., Mayadas T.N. (2012). Endocytosis of soluble immune complexes leads to their clearance by fcgammariiib but induces neutrophil extracellular traps via fcgammariia in vivo. Blood.

[93] Wang Z., Li J., Cho J., Malik A.B. (2014). Prevention of vascular inflammation by nanoparticle targeting of adherent neutrophils. Nat. Nanotechnol..

[94] Fabriek B.O., Dijkstra C.D., van den Berg T.K. (2005). The macrophage scavenger receptor cd163. Immunobiology.

[95] Andersen M.N., Etzerodt A., Graversen J.H., Holthof L.C., Moestrup S.K., Hokland M., Moller H.J. (2019). Stat3 inhibition specifically in human monocytes and macrophages by cd163-targeted corosolic acid-containing liposomes. Cancer Immunol. Immunother. CII.

[96] Suzuki Y., Shirai M., Asada K., Yasui H., Karayama M., Hozumi H., Furuhashi K., Enomoto N., Fujisawa T., Nakamura Y. (2018). Macrophage mannose receptor, cd206, predict prognosis in patients with pulmonary tuberculosis. Sci. Rep..

[97] Song Y., Tang C., Yin C. (2018). Combination antitumor immunotherapy with vegf and pigf sirna via systemic delivery of multi-functionalized nanoparticles to tumor-associated macrophages and breast cancer cells. Biomaterials.

[98] Puig-Kroger A., Sierra-Filardi E., Dominguez-Soto A., Samaniego R., Corcuera M.T., Gomez-Aguado F., Ratnam M., Sanchez-Mateos P., Corbi A.L. (2009). Folate receptor beta is expressed by tumor-associated macrophages and constitutes a marker for m2 anti-inflammatory/regulatory macrophages. Cancer Res..

[99] Soto E.R., Caras A.C., Kut L.C., Castle M.K., Ostroff G.R. (2012). Glucan particles for macrophage targeted delivery of nanoparticles. J. Drug Deliv..

[100] Song X., Wan Z., Chen T., Fu Y., Jiang K., Yi X., Ke H., Dong J., Yang L., Li L. (2016). Development of a multi-target peptide for potentiating chemotherapy by modulating tumor microenvironment. Biomaterials.

[101] Tang C., Wang C., Zhang Y., Xue L., Li Y., Ju C., Zhang C. (2019). Recognition, intervention, and monitoring of neutrophils in acute ischemic stroke. Nano Lett..

[102] Dong X., Gao J., Zhang C.Y., Hayworth C., Frank M., Wang Z. (2019). Neutrophil membrane-derived nanovesicles alleviate inflammation to protect mouse brain injury from ischemic stroke. ACS Nano.

[103] Leuschner F., Dutta P., Gorbatov R., Novobrantseva T.I., Donahoe J.S., Courties G., Lee K.M., Kim J.I., Markmann J.F., Marinelli B. (2011). Therapeutic sirna silencing in inflammatory monocytes in mice. Nat. Biotechnol..

[104] Robertson J.D., Ward J.R., Avila-Olias M., Battaglia G., Renshaw S.A. (2017). Targeting neutrophilic inflammation using polymersome-mediated cellular delivery. J. Immunol..

[105] Zhang C.Y., Dong X., Gao J., Lin W., Liu Z., Wang Z. (2019). Nanoparticle-induced neutrophil apoptosis increases survival in sepsis and alleviates neurological damage in stroke. Sci. Adv..

[106] Van Rooijen N., Sanders A. (1994). Liposome mediated depletion of macrophages: Mechanism of action, preparation of liposomes and applications. J. Immunol. Methods.

[107] Van Lent P.L., Holthuysen A.E., Van Rooijen N., Van De Putte L.B., Van Den Berg W.B. (1998). Local removal of phagocytic synovial lining cells by clodronate-liposomes decreases cartilage destruction during collagen type ii arthritis. Ann. Rheum. Dis..

[108] Barrera P., Blom A., van Lent P.L., van Bloois L., Beijnen J.H., van Rooijen N., de Waal Malefijt M.C., van de Putte L.B., Storm G., van den Berg W.B. (2000). Synovial macrophage depletion with clodronate-containing liposomes in rheumatoid arthritis. Arthritis Rheum..

[109] Waltl I., Kaufer C., Broer S., Chhatbar C., Ghita L., Gerhauser I., Anjum M., Kalinke U., Loscher W. (2018). Macrophage depletion by liposome-encapsulated clodronate suppresses seizures but not hippocampal damage after acute viral encephalitis. Neurobiol. Dis..

[110] Griesmann H., Drexel C., Milosevic N., Sipos B., Rosendahl J., Gress T.M., Michl P. (2017). Pharmacological macrophage inhibition decreases metastasis formation in a genetic model of pancreatic cancer. Gut.

[111] Fink K., Ng C., Nkenfou C., Vasudevan S.G., van Rooijen N., Schul W. (2009). Depletion of macrophages in mice results in higher dengue virus titers and highlights the role of macrophages for virus control. Eur. J. Immunol..

[112] Chakravarthy K.V., Davidson B.A., Helinski J.D., Ding H., Law W.C., Yong K.T., Prasad P.N., Knight P.R. (2011). Doxorubicin-conjugated quantum dots to target alveolar macrophages and inflammation. Nanomed. Nanotechnol. Biol. Med..

[113] Qindeel M., Khan D., Ahmed N., Khan S., Asim Ur R. (2020). Surfactant-free, self-assembled nanomicelles-based transdermal hydrogel for safe and targeted delivery of methotrexate against rheumatoid arthritis. ACS Nano.

[114] Tang J., Lobatto M.E., Hassing L., van der Staay S., van Rijs S.M., Calcagno C., Braza M.S., Baxter S., Fay F., Sanchez-Gaytan B.L. (2015). Inhibiting macrophage proliferation suppresses atherosclerotic plaque inflammation. Sci. Adv..

[115] Zhang P., Miska J., Lee-Chang C., Rashidi A., Panek W.K., An S., Zannikou M., Lopez-Rosas A., Han Y., Xiao T. (2019). Therapeutic targeting of tumor-associated myeloid cells synergizes with radiation therapy for glioblastoma. Proc. Natl. Acad. Sci. USA.

[116] Zang X., Zhang X., Hu H., Qiao M., Zhao X., Deng Y., Chen D. (2019). Targeted delivery of zoledronate to tumor-associated macrophages for cancer immunotherapy. Mol. Pharm..

[117] Nagaraj S., Gabrilovich D.I. (2007). Myeloid-derived suppressor cells. Adv. Exp. Med. Biol..

[118] Fridlender Z.G., Sun J., Kim S., Kapoor V., Cheng G., Ling L., Worthen G.S., Albelda S.M. (2009). Polarization of tumor-associated neutrophil phenotype by TGF-β: “N1” versus “N2” tan. Cancer Cell.

[119] King I.L., Dickendesher T.L., Segal B.M. (2009). Circulating ly-6C^+^ myeloid precursors migrate to the cns and play a pathogenic role during autoimmune demyelinating disease. Blood.

[120] Westendorf A.M., Fleissner D., Deppenmeier S., Gruber A.D., Bruder D., Hansen W., Liblau R., Buer J. (2006). Autoimmune-mediated intestinal inflammation-impact and regulation of antigen-specific cd8^+^ T cells. Gastroenterology.

[121] Marhaba R., Vitacolonna M., Hildebrand D., Baniyash M., Freyschmidt-Paul P., Zoller M. (2007). The importance of myeloid-derived suppressor cells in the regulation of autoimmune effector cells by a chronic contact eczema. J. Immunol..

[122] Buckley C.D., Ross E.A., McGettrick H.M., Osborne C.E., Haworth O., Schmutz C., Stone P.C., Salmon M., Matharu N.M., Vohra R.K. (2006). Identification of a phenotypically and functionally distinct population of long-lived neutrophils in a model of reverse endothelial migration. J. Leukoc. Biol..

[123] Fites J.S., Gui M., Kernien J.F., Negoro P., Dagher Z., Sykes D.B., Nett J.E., Mansour M.K., Klein B.S. (2018). An unappreciated role for neutrophil-dc hybrids in immunity to invasive fungal infections. PLoS Pathog..

[124] Singhal S., Bhojnagarwala P.S., O’Brien S., Moon E.K., Garfall A.L., Rao A.S., Quatromoni J.G., Stephen T.L., Litzky L., Deshpande C. (2016). Origin and role of a subset of tumor-associated neutrophils with antigen-presenting cell features in early-stage human lung cancer. Cancer Cell.

[125] Andzinski L., Kasnitz N., Stahnke S., Wu C.F., Gereke M., von Kockritz-Blickwede M., Schilling B., Brandau S., Weiss S., Jablonska J. (2016). Type I ifns induce anti-tumor polarization of tumor associated neutrophils in mice and human. Int. J. Cancer.

[126] Ma Y., Yabluchanskiy A., Iyer R.P., Cannon P.L., Flynn E.R., Jung M., Henry J., Cates C.A., Deleon-Pennell K.Y., Lindsey M.L. (2016). Temporal neutrophil polarization following myocardial infarction. Cardiovasc. Res..

[127] Pillay J., Ramakers B.P., Kamp V.M., Loi A.L., Lam S.W., Hietbrink F., Leenen L.P., Tool A.T., Pickkers P., Koenderman L. (2010). Functional heterogeneity and differential priming of circulating neutrophils in human experimental endotoxemia. J. Leukoc. Biol..

[128] Pillay J., Kamp V.M., van Hoffen E., Visser T., Tak T., Lammers J.W., Ulfman L.H., Leenen L.P., Pickkers P., Koenderman L. (2012). A subset of neutrophils in human systemic inflammation inhibits T cell responses through mac-1. J. Clin. Investig..

[129] Hacbarth E., Kajdacsy-Balla A. (1986). Low density neutrophils in patients with systemic lupus erythematosus, rheumatoid arthritis, and acute rheumatic fever. Arthritis Rheum..

[130] Yang H., Fung S.Y., Xu S., Sutherland D.P., Kollmann T.R., Liu M., Turvey S.E. (2015). Amino acid-dependent attenuation of toll-like receptor signaling by peptide-gold nanoparticle hybrids. ACS Nano.

[131] Yang H., Kozicky L., Saferali A., Fung S.Y., Afacan N., Cai B., Falsafi R., Gill E., Liu M., Kollmann T.R. (2016). Endosomal ph modulation by peptide-gold nanoparticle hybrids enables potent anti-inflammatory activity in phagocytic immune cells. Biomaterials.

[132] Yang H., Fung S.Y., Bao A., Li Q., Turvey S.E. (2017). Screening bioactive nanoparticles in phagocytic immune cells for inhibitors of toll-like receptor signaling. J. Vis. Exp. JoVE.

[133] Wang L., Zhang H., Sun L., Gao W., Xiong Y., Ma A., Liu X., Shen L., Li Q., Yang H. (2020). Manipulation of macrophage polarization by peptide-coated gold nanoparticles and its protective effects on acute lung injury. J. Nanobiotechnol..

[134] Raimondo T.M., Mooney D.J. (2018). Functional muscle recovery with nanoparticle-directed m2 macrophage polarization in mice. Proc. Natl. Acad. Sci. USA.

[135] Alvarado-Vazquez P.A., Bernal L., Paige C.A., Grosick R.L., Moracho Vilrriales C., Ferreira D.W., Ulecia-Moron C., Romero-Sandoval E.A. (2017). Macrophage-specific nanotechnology-driven cd163 overexpression in human macrophages results in an m2 phenotype under inflammatory conditions. Immunobiology.

[136] Kang H., Zhang K., Wong D.S.H., Han F., Li B., Bian L. (2018). Near-infrared light-controlled regulation of intracellular calcium to modulate macrophage polarization. Biomaterials.

[137] Kraynak C.A., Yan D.J., Suggs L.J. (2020). Modulating inflammatory macrophages with an apoptotic body-inspired nanoparticle. Acta Biomater..

[138] Gao Q., Zhang J., Chen C., Chen M., Sun P., Du W., Zhang S., Liu Y., Zhang R., Bai M. (2020). In situ mannosylated nanotrinity-mediated macrophage remodeling combats candida albicans infection. ACS Nano.

[139] Sollberger G., Choidas A., Burn G.L., Habenberger P., Di Lucrezia R., Kordes S., Menninger S., Eickhoff J., Nussbaumer P., Klebl B. (2018). Gasdermin d plays a vital role in the generation of neutrophil extracellular traps. Sci. Immunol..

[140] Chen K.W., Monteleone M., Boucher D., Sollberger G., Ramnath D., Condon N.D., von Pein J.B., Broz P., Sweet M.J., Schroder K. (2018). Noncanonical inflammasome signaling elicits gasdermin d-dependent neutrophil extracellular traps. Sci. Immunol..

[141] Jorch S.K., Kubes P. (2017). An emerging role for neutrophil extracellular traps in noninfectious disease. Nat. Med..

[142] Daniel C., Leppkes M., Munoz L.E., Schley G., Schett G., Herrmann M. (2019). Extracellular DNA traps in inflammation, injury and healing. Nat. Rev. Nephrol..

[143] Campbell A.M., Kashgarian M., Shlomchik M.J. (2012). Nadph oxidase inhibits the pathogenesis of systemic lupus erythematosus. Sci. Transl. Med..

[144] Cedervall J., Zhang Y., Huang H., Zhang L., Femel J., Dimberg A., Olsson A.K. (2015). Neutrophil extracellular traps accumulate in peripheral blood vessels and compromise organ function in tumor-bearing animals. Cancer Res..

[145] Okeke E.B., Louttit C., Fry C., Najafabadi A.H., Han K., Nemzek J., Moon J.J. (2020). Inhibition of neutrophil elastase prevents neutrophil extracellular trap formation and rescues mice from endotoxic shock. Biomaterials.

[146] Spence S., Greene M.K., Fay F., Hams E., Saunders S.P., Hamid U., Fitzgerald M., Beck J., Bains B.K., Smyth P. (2015). Targeting siglecs with a sialic acid-decorated nanoparticle abrogates inflammation. Sci. Transl. Med..

[147] Bornhofft K.F., Viergutz T., Kuhnle A., Galuska S.P. (2019). Nanoparticles equipped with α2,8-linked sialic acid chains inhibit the release of neutrophil extracellular traps. Nanomaterials.

[148] Laroui H., Theiss A.L., Yan Y., Dalmasso G., Nguyen H.T., Sitaraman S.V., Merlin D. (2011). Functional TNFα gene silencing mediated by polyethyleneimine/ TNFα sirna nanocomplexes in inflamed colon. Biomaterials.

[149] Xiao B., Laroui H., Ayyadurai S., Viennois E., Charania M.A., Zhang Y., Merlin D. (2013). Mannosylated bioreducible nanoparticle-mediated macrophage-specific TNF-α RNA interference for IBD therapy. Biomaterials.

[150] Kosovrasti V.Y., Nechev L.V., Amiji M.M. (2016). Peritoneal macrophage-specific TNF-α gene silencing in LPS-induced acute inflammation model using CD44 targeting hyaluronic acid nanoparticles. Mol. Pharm..

[151] Jiang Y., Hardie J., Liu Y., Ray M., Luo X., Das R., Landis R.F., Farkas M.E., Rotello V.M. (2018). Nanocapsule-mediated cytosolic sirna delivery for anti-inflammatory treatment. J. Control. Release.

[152] Duan B., Li M., Sun Y., Zou S., Xu X. (2019). Orally delivered antisense oligodeoxyribonucleotides of TNF-α via polysaccharide-based nanocomposites targeting intestinal inflammation. Adv. Healthc. Mater..

[153] Xiao B., Chen Q., Zhang Z., Wang L., Kang Y., Denning T., Merlin D. (2018). Tnfα gene silencing mediated by orally targeted nanoparticles combined with interleukin-22 for synergistic combination therapy of ulcerative colitis. J. Control. Release.

[154] Peng L., Chen X. (2015). Antibody-drug conjugates. Bioconjug. Chem..

[155] Luo Y.L., Xu C.F., Li H.J., Cao Z.T., Liu J., Wang J.L., Du X.J., Yang X.Z., Gu Z., Wang J. (2018). Macrophage-specific in vivo gene editing using cationic lipid-assisted polymeric nanoparticles. ACS Nano.

[156] Coffelt S.B., Kersten K., Doornebal C.W., Weiden J., Vrijland K., Hau C.S., Verstegen N.J.M., Ciampricotti M., Hawinkels L., Jonkers J. (2015). IL-17-producing γβ T cells and neutrophils conspire to promote breast cancer metastasis. Nature.

[157] Wu P., Wu D., Ni C., Ye J., Chen W., Hu G., Wang Z., Wang C., Zhang Z., Xia W. (2014). Gammadeltat17 cells promote the accumulation and expansion of myeloid-derived suppressor cells in human colorectal cancer. Immunity.

[158] Nywening T.M., Belt B.A., Cullinan D.R., Panni R.Z., Han B.J., Sanford D.E., Jacobs R.C., Ye J., Patel A.A., Gillanders W.E. (2018). Targeting both tumour-associated CXCR2^+^ neutrophils and CCR2^+^ macrophages disrupts myeloid recruitment and improves chemotherapeutic responses in pancreatic ductal adenocarcinoma. Gut.

[159] Schott A.F., Goldstein L.J., Cristofanilli M., Ruffini P.A., McCanna S., Reuben J.M., Perez R.P., Kato G., Wicha M. (2017). Phase ib pilot study to evaluate reparixin in combination with weekly paclitaxel in patients with her-2-negative metastatic breast cancer. Clin. Cancer Res..

[160] Jaillon S., Ponzetta A., Di Mitri D., Santoni A., Bonecchi R., Mantovani A. (2020). Neutrophil diversity and plasticity in tumour progression and therapy. Nat. Rev. Cancer.

[161] Chu D., Dong X., Shi X., Zhang C., Wang Z. (2018). Neutrophil-based drug delivery systems. Adv. Mater..

[162] Dong X., Chu D., Wang Z. (2017). Leukocyte-mediated delivery of nanotherapeutics in inflammatory and tumor sites. Theranostics.

[163] Dong X., Chu D., Wang Z. (2018). Neutrophil-mediated delivery of nanotherapeutics across blood vessel barrier. Ther. Deliv..

[164] Sapey E., Greenwood H., Walton G., Mann E., Love A., Aaronson N., Insall R.H., Stockley R.A., Lord J.M. (2014). Phosphoinositide 3-kinase inhibition restores neutrophil accuracy in the elderly: Toward targeted treatments for immunosenescence. Blood.

[165] Alves-Filho J.C., Sonego F., Souto F.O., Freitas A., Verri W.A., Auxiliadora-Martins M., Basile-Filho A., McKenzie A.N., Xu D., Cunha F.Q. (2010). Interleukin-33 attenuates sepsis by enhancing neutrophil influx to the site of infection. Nat. Med..

[166] Yen C.L., Liao Y.C., Chen R.F., Huang Y.F., Chung W.C., Lo P.C., Chang C.F., Wu P.C., Shieh D.B., Jiang S.T. (2019). Targeted delivery of curcumin rescues endoplasmic reticulum-retained mutant nox2 protein and avoids leukocyte apoptosis. J. Immunol..

[167] DeNardo D.G., Ruffell B. (2019). Macrophages as regulators of tumour immunity and immunotherapy. Nat. Rev. Immunol..

[168] Ramesh A., Kumar S., Nandi D., Kulkarni A. (2019). Csf1r- and shp2-inhibitor-loaded nanoparticles enhance cytotoxic activity and phagocytosis in tumor-associated macrophages. Adv. Mater..

[169] Bose R.J.C., Tharmalingam N., Garcia Marques F.J., Sukumar U.K., Natarajan A., Zeng Y., Robinson E., Bermudez A., Chang E., Habte F. (2020). Reconstructed apoptotic bodies as targeted “nano decoys” to treat intracellular bacterial infections within macrophages and cancer cells. ACS Nano.

[170] Chu D., Gao J., Wang Z. (2015). Neutrophil-mediated delivery of therapeutic nanoparticles across blood vessel barrier for treatment of inflammation and infection. ACS Nano.

[171] Zhang C., Ling C.L., Pang L., Wang Q., Liu J.X., Wang B.S., Liang J.M., Guo Y.Z., Qin J., Wang J.X. (2017). Direct macromolecular drug delivery to cerebral ischemia area using neutrophil-mediated nanoparticles. Theranostics.

[172] Hou J., Yang X., Li S., Cheng Z., Wang Y., Zhao J., Zhang C., Li Y., Luo M., Ren H. (2019). Accessing neuroinflammation sites: Monocyte/neutrophil-mediated drug delivery for cerebral ischemia. Sci. Adv..

[173] Zhang W., Wang M., Tang W., Wen R., Zhou S., Lee C., Wang H., Jiang W., Delahunty I.M., Zhen Z. (2018). Nanoparticle-laden macrophages for tumor-tropic drug delivery. Adv. Mater..

[174] Miller M.A., Zheng Y.R., Gadde S., Pfirschke C., Zope H., Engblom C., Kohler R.H., Iwamoto Y., Yang K.S., Askevold B. (2015). Tumour-associated macrophages act as a slow-release reservoir of nano-therapeutic pt(IV) pro-drug. Nat. Commun..

[175] Xue Y., Wu Y., Wang Q., Xue L., Su Z., Zhang C. (2019). Cellular vehicles based on neutrophils enable targeting of atherosclerosis. Mol. Pharm..

[176] Hu L., Luo X., Zhou S., Zhu J., Xiao M., Li C., Zheng H., Qiu Q., Lai C., Liu X. (2019). Neutrophil-mediated delivery of dexamethasone palmitate-loaded liposomes decorated with a sialic acid conjugate for rheumatoid arthritis treatment. Pharm. Res..

[177] Doshi N., Swiston A.J., Gilbert J.B., Alcaraz M.L., Cohen R.E., Rubner M.F., Mitragotri S. (2011). Cell-based drug delivery devices using phagocytosis-resistant backpacks. Adv. Mater..

[178] Anselmo A.C., Gilbert J.B., Kumar S., Gupta V., Cohen R.E., Rubner M.F., Mitragotri S. (2015). Monocyte-mediated delivery of polymeric backpacks to inflamed tissues: A generalized strategy to deliver drugs to treat inflammation. J. Control. Release.

[179] Klyachko N.L., Polak R., Haney M.J., Zhao Y., Gomes Neto R.J., Hill M.C., Kabanov A.V., Cohen R.E., Rubner M.F., Batrakova E.V. (2017). Macrophages with cellular backpacks for targeted drug delivery to the brain. Biomaterials.

[180] Shields C.W., Evans M.A., Wang L.L., Baugh N., Iyer S., Wu D., Zhao Z., Pusuluri A., Ukidve A., Pan D.C. (2020). Cellular backpacks for macrophage immunotherapy. Sci. Adv..

[181] Shirasuna K., Karasawa T., Takahashi M. (2019). Exogenous nanoparticles and endogenous crystalline molecules as danger signals for the nlrp3 inflammasomes. J. Cell. Physiol..

[182] Vita A.A., Royse E.A., Pullen N.A. (2019). Nanoparticles and danger signals: Oral delivery vehicles as potential disruptors of intestinal barrier homeostasis. J. Leukoc. Biol..

[183] Gallud A., Fadeel B. (2015). Keeping it small: Towards a molecular definition of nanotoxicology. Eur. J. Nanomed..

[184] Mukherjee S.P., Bondarenko O., Kohonen P., Andon F.T., Brzicova T., Gessner I., Mathur S., Bottini M., Calligari P., Stella L. (2018). Macrophage sensing of single-walled carbon nanotubes via toll-like receptors. Sci. Rep..

[185] Turabekova M., Rasulev B., Theodore M., Jackman J., Leszczynska D., Leszczynski J. (2014). Immunotoxicity of nanoparticles: A computational study suggests that cnts and c60 fullerenes might be recognized as pathogens by toll-like receptors. Nanoscale.

[186] Fraser J.A., Kemp S., Young L., Ross M., Prach M., Hutchison G.R., Malone E. (2018). Silver nanoparticles promote the emergence of heterogeneic human neutrophil sub-populations. Sci. Rep..

[187] Muzi L., Tardani F., La Mesa C., Bonincontro A., Bianco A., Risuleo G. (2016). Interactions and effects of bsa-functionalized single-walled carbon nanotubes on different cell lines. Nanotechnology.

[188] Desai J., Foresto-Neto O., Honarpisheh M., Steiger S., Nakazawa D., Popper B., Buhl E.M., Boor P., Mulay S.R., Anders H.J. (2017). Particles of different sizes and shapes induce neutrophil necroptosis followed by the release of neutrophil extracellular trap-like chromatin. Sci. Rep..

[189] Munoz L.E., Bilyy R., Biermann M.H., Kienhofer D., Maueroder C., Hahn J., Brauner J.M., Weidner D., Chen J., Scharin-Mehlmann M. (2016). Nanoparticles size-dependently initiate self-limiting netosis-driven inflammation. Proc. Natl. Acad. Sci. USA.

[190] Getts D.R., Terry R.L., Getts M.T., Deffrasnes C., Muller M., van Vreden C., Ashhurst T.M., Chami B., McCarthy D., Wu H. (2014). Therapeutic inflammatory monocyte modulation using immune-modifying microparticles. Sci. Transl. Med..

[191] Jeong S.J., Cooper J.G., Ifergan I., McGuire T.L., Xu D., Hunter Z., Sharma S., McCarthy D., Miller S.D., Kessler J.A. (2017). Intravenous immune-modifying nanoparticles as a therapy for spinal cord injury in mice. Neurobiol. Dis..

[192] Saito E., Kuo R., Pearson R.M., Gohel N., Cheung B., King N.J.C., Miller S.D., Shea L.D. (2019). Designing drug-free biodegradable nanoparticles to modulate inflammatory monocytes and neutrophils for ameliorating inflammation. J. Control. Release.

[193] MacParland S.A., Tsoi K.M., Ouyang B., Ma X.Z., Manuel J., Fawaz A., Ostrowski M.A., Alman B.A., Zilman A., Chan W.C. (2017). Phenotype determines nanoparticle uptake by human macrophages from liver and blood. ACS Nano.

[194] Wang Z. (2016). Imaging nanotherapeutics in inflamed vasculature by intravital microscopy. Theranostics.

